# The Impact of Hydrogen Peroxide (H_2_O_2_) Fumigation on Bacterial Levels in Dental Office Environments: A Randomized Clinical Trial Investigation

**DOI:** 10.3390/jcm12247551

**Published:** 2023-12-07

**Authors:** Jacek Matys, Tomasz Gedrange, Marzena Dominiak, Kinga Grzech-Leśniak

**Affiliations:** 1 Oral Surgery Department, Wroclaw Medical University, 50-425 Wroclaw, Poland; tomasz.gedrange@umw.edu.pl (T.G.); marzena.dominiak@umw.edu.pl (M.D.); kinga.grzech-lesniak@umw.edu.pl (K.G.-L.); 2 Department of Orthodontics, Technische Universitat Dresden, 01307 Dresden, Germany; 3 Department of Periodontics, School of Dentistry, Virginia Commonwealth University, Richmond, VA 23284, USA

**Keywords:** aerosol, antibacterial, bacteria, vapor

## Abstract

Background: Fumigation with hydrogen peroxide has proven to be a highly effective approach to maintaining biological safety within dental offices. The main purpose of this research was to investigate the efficacy of hydrogen peroxide (H_2_O_2_) fumigation in reducing bacterial levels in dental office environments. Methods: The study involved 30 participants diagnosed with moderate caries decay (ICDAS 3 and 4) in their mandibular molars. Sixty Petri dishes (two per patient) with Columbia Agar and 5% Sheep Blood were opened at the beginning of the caries treatment. After the completion of caries treatment and tooth restoration, 30 plates (G1 group) were closed. Following this, a 20 min fumigation procedure with 6% hydrogen peroxide biosanitizer using a compressed air device was conducted. After the fumigation, the remaining plates were closed (G2 group). The total number of bacteria CFUs (colony-forming units) in the dental office air was determined using the Koch sedimentation method. Results: The total bacterial colony (TBC) level, measured in cfu/m^3^, demonstrated a significant decrease in the number of bacteria following room environment fumigation (163.1 ± 145.7; G2 group) compared to non-fumigated samples (817.2 ± 208.2; G1 group) (*p* < 0.001). The predominant bacteria observed in the microbiological plates before fumigation were *Micrococcus* and *Bacillus* species, found in 80% (24/30) and 60% (18/30) of the plates, respectively. Application of H2O2 room fumigation resulted in a significant reduction in bacterial numbers: 79.2% (5/30) for *Micrococcus* species (*p* < 0.001), 83.3% (3/30) for *Bacillus* species (*p* < 0.001), and 100% (0/30) for *Staphylococcus arlettae* (*p* < 0.05). Conclusion: Fumigation with 6% H_2_O_2_ is an effective method for reducing bacterial counts in a dental office environment.

## 1. Introduction

The decontamination of dental office environments is of paramount importance in ensuring a safe and sanitary setting for both dental practitioners and patients [1,2]. Cross-contamination, particularly through the presence of microbial species, poses a significant risk within the dental office [3,4]. The thorough investigation of microbial communities, especially those cultured from surface swabs, was essential in understanding the potential sources of contamination [5,6]. Microbes, such as bacteria, viruses, and fungi, are ubiquitous in the dental setting and can persist on surfaces, instruments, and in the air [7]. Identifying the most prevalent microbial species and assessing their abundance provides valuable insights into the effectiveness of decontamination procedures and the potential for cross-contamination [3]. Bacteria, commonly found in the oral cavity and on various surfaces, may contribute to the risk of nosocomial infections if not adequately controlled [4]. Furthermore, recently published studies of microbial species in dental office environments have helped us to understand the dynamics of these microorganisms and their potential role in the transmission of infections in healthcare facilities [8,9].

Hydrogen peroxide fumigation, commonly known as hydrogen peroxide vapor (HPV) sterilization, stands as a highly effective method for ensuring biological safety in dental offices [10,11,12]. By denaturing proteins and enzymes, hydrogen peroxide vapor effectively eliminates a wide range of microorganisms, including bacteria, viruses, fungi, and spores [13]. One of this vapor’s key advantages lies in its ability to penetrate intricate surfaces and hard-to-reach areas within the dental office, ensuring a comprehensive disinfection process [7]. Crucially, it leaves no harmful residues, breaking down into water and oxygen, making it a residue-free and environmentally friendly option [11]. However, proper safety measures, including the use of personal protective equipment and ventilation, are essential during the fumigation process [1,14,15,16].

To maintain biological safety, dental professionals need to be aware of the compatibility of materials and equipment with hydrogen peroxide fumigation [17,18]. Sensitive materials might degrade over time, necessitating careful assessment before fumigation [7]. Additionally, electronic devices are particularly susceptible to damage, requiring special protection or alternative disinfection methods [7]. Regular verification and monitoring through biological indicators and chemical strips are crucial to confirm the sterilization’s effectiveness. Adhering to guidelines and consulting with experts in infection control and occupational safety ensures that hydrogen peroxide fumigation in dental offices not only meets high standards of biological safety but also provides a secure environment for both patients and dental healthcare workers [1,7,18].

Hydrogen peroxide exerts its antimicrobial effects on bacterial cells through oxidative stress mechanisms [11,18]. Upon penetration of the cell membrane, hydrogen peroxide generates reactive oxygen species (ROS) like hydroxyl radicals and superoxide radicals, which induce oxidative stress by disrupting the cellular redox balance [19]. ROS target proteins, causing conformational changes and functional loss, particularly in vital enzymes, interfering with bacterial metabolism [20]. Additionally, hydrogen peroxide induces DNA damage, including strand breaks and base modifications, disrupting replication and transcription processes [21]. Lipid peroxidation compromises cell membrane integrity, increasing permeability and disrupting ion gradients, which are essential to cellular functions. Enzyme inactivation due to the release of metal cofactors disrupts metabolic pathways [20]. Direct disruption of the lipid bilayer and subsequent leakage of cellular contents cause bacterial cell lysis. These multifaceted actions collectively lead to bacterial cell death, highlighting hydrogen peroxide’s significance as an antimicrobial agent in various applications [19,20,21,22].

In dental offices, hydrogen peroxide fumigation is a meticulous process involving several steps to ensure effective disinfection [1,17]. First, the area to be treated is sealed off, ensuring a contained environment [1]. The hydrogen peroxide solution is then vaporized using specialized equipment, creating a fine mist of hydrogen peroxide vapor that permeates the entire space [12,14]. This vapor phase is crucial as it can reach and disinfect even hard-to-access areas. The concentration and exposure time are closely monitored to guarantee effectiveness while ensuring the safety of both patients and dental staff. After a specified duration, aeration takes place, allowing the hydrogen peroxide vapor to naturally dissipate and break down into harmless water and oxygen molecules [1,23]. During the entire process, stringent safety protocols, including the use of personal protective equipment and proper ventilation, are followed to protect everyone involved. Regular monitoring and verification, often using biological and chemical indicators, confirm the efficacy of the fumigation, ensuring a thoroughly disinfected and biologically safe dental environment [1,12,24].

The main purpose of this research was to investigate the efficacy of hydrogen peroxide (H_2_O_2_) fumigation in reducing bacterial levels in dental office environments. The study aimed to test the null hypothesis that there was no significant difference in bacterial counts before and after the implementation of H_2_O_2_ fumigation. The research specifically focused on the impact of fumigation on the total bacterial colony levels and the prevalence of specific bacterial species in the dental office air. Also, the findings aimed to provide insights into the effectiveness of H_2_O_2_ as a biosanitizer for maintaining biological safety in dental settings.

## 2. Materials and Methods

The study was carried out as a randomized and controlled trial. Before initiation, approval was obtained from the Local Ethics Committee of the Faculty of Dentistry at Wroclaw Medical University (permission number: KB-737/2021), and all participants gave informed consent following the principles of the Helsinki Declaration. The clinical trial was registered with ClinicalTrials.gov accessed on 29 October 2023 (identifier: NCT06100848).

### 2.1. Participants

**Figure 1:**
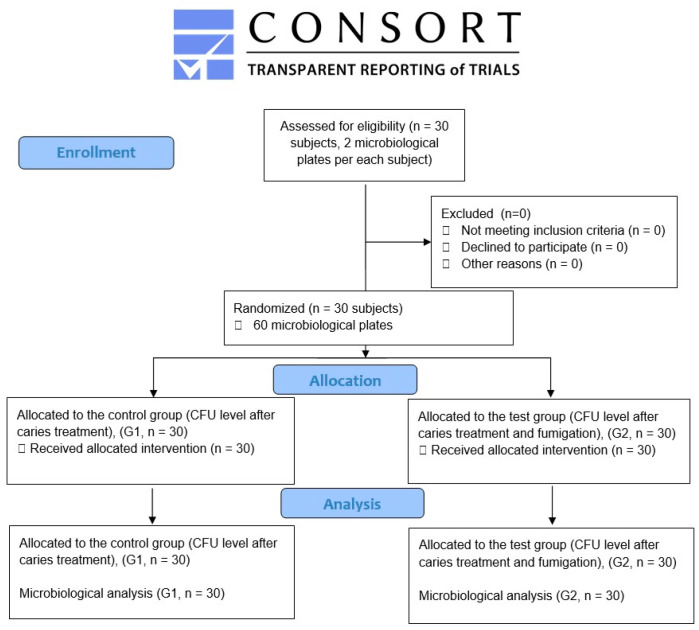
The diagram illustrates the subjects’ treatment following the CONSORT 2010 guidelines.

The study included 30 participants, consisting of 18 females and 12 males, all diagnosed with moderate caries decay based on the International Caries Detection and Assessment System (ICDAS 3 and 4) in their mandibular molar teeth. These participants had an average age of 42.2 ± 8.3 years. The sample size of 30 microbiological plates with Columbia Agar and 5% Sheep Blood in each group was calculated using the G*Power software (Version 3.1) from Kiel University, Germany, considering our prior research [14,16], with a significance level of 0.05, an effect size (d) of 0.72, a 95% confidence interval, and 85% power. Stringent inclusion criteria were applied, ensuring participants did not use anti-inflammatory medications, were non-smokers, had no systemic illnesses, and had not taken antibiotics in the last two months. Additionally, they did not have uncompensated diabetes or uncontrolled periodontal disease, exhibited no halitosis symptoms, had no gastric diseases, maintained good oral hygiene, and had received hygienist treatment two weeks before the study initiation. The study was designed in accordance with the CONSORT 2010 guidelines [25] (Figure 1). 

### 2.2. Caries Treatment Procedure and Study Design

Prior to caries treatment, a rubber dam was placed in the patient’s mouth to ensure precise isolation. Caries treatment was administered to all patients using a round diamond bur (#014) in combination with a W&H Synea TA-98LC high-speed handpiece (W&H, Bürmoos, Austria) operating at 200,000 RPM (revolutions per minute) and water-cooling set at 30 mL/min. To maintain a clean environment, a conventional high-volume evacuator (HVE) EM19 EVO (Monoart^®^ Euronda, Vicenza, Italy) was utilized to effectively remove aerosols during the procedure. The caries preparation took an average of 60 s per tooth. The treatment was conducted in accordance with common dental standards for the treatment and restoration of tooth cavities. Following preparation, the teeth were meticulously restored using composite materials (Charisma Classic, Kulzer, Hanau, Germany), then the finishing and polishing procedure for the restoration was carried out. Simultaneously with the commencement of caries treatment, two plates containing a microbiological culture medium (Columbia Agar and 5% Sheep Blood) were opened for each patient. After completing the patient’s treatment, even if the procedure took less than 30 min (with the average visit duration being 27 min), the samples were exposed for a total of 40 min from the beginning of the patient’s visit when the microbiological plates were initially opened. Subsequently, one of the samples was sealed, while the other remained open until the dental office room was fumigated. A coin toss was performed to randomly choose which plates would undergo fumigation. The plates were positioned at a height of 1 m from the ground and 2 m away from the patient’s mouth. The fumigation process of the dental office environment began immediately after one of the two opened plates was closed and removed from the room.

### 2.3. Standardizing Surfaces and Air Quality in the Dental Office

Following each patient’s visit, a meticulous cleaning protocol was initiated, targeting key areas like dental chairs, medical equipment, and handles. Utilizing a disinfectant cleaner, surfaces were carefully wiped down to eliminate visible debris. The aspiration system underwent a comprehensive cleaning regimen, including the passage of a cleaning solution to clear debris and disinfect internal components. Concurrently, suction lines were disinfected to uphold optimal functionality. External surfaces were treated with disinfectant, allowing a resting period of ten minutes. The dental office, spanning a twenty-square-meter area, underwent a thorough series of standardization procedures. This involved the secure closure of all windows and doors, coupled with the deactivation of the air conditioning system. To sustain aerosol levels within the specified range of 28,000 to 30,000 particles per cubic meter during procedures, the NV1050 (Novaerus, Dublin, Ireland) air purifier, boasting an air exchange rate of 800 m^3^ per hour, was deployed. Continuous monitoring at one-minute intervals occurred throughout the air purifier’s operation. Dental treatments were exclusively conducted after achieving meticulous air standardization within the designated range. Control measurements, acquired via a particle sensor PM200 (Trotec GmbH, Schwerin, Germany) at the office’s central point, revealed an average duration of approximately five minutes to thoroughly cleanse the air to the specified levels [15].

### 2.4. Microbial Analysis

The Koch sedimentation method was utilized to assess the aerobic bacterial content in the air of the dental office [26]. After 48 h of incubation at 37 °C, the degree of microbiological contamination was determined, calculated as the total number of CFUs (colony-forming units) in one cubic meter of air using the formula: L = a × 1000/(πr^2^ × k). In the formula, ‘L’ represents the microbial contamination level in [cfu/m^3^], ‘a’ represents the quantity of bacterial colonies cultivated on the plate, ‘r’ represents the Petri dish radius [cm], and ‘k’ represents the plate exposure time factor where k = t × 1/5, and ‘t’ represents the exposure time in minutes. During the first phase, bacterial identification was performed according to standard protocols, involving microscopic observation of morphology, Gram staining, and evaluation of size, shape, and spore presence. In the second phase, bacterial metabolism was analyzed using the APIWeb-supported Analytical Profile Index (API) by Biomerioux Inc. (Durham, NC, USA). Quality control procedures were adhered to in accordance with the PN-EN12322 standard [27].

### 2.5. Fumigation Procedure

**Figure 2:**
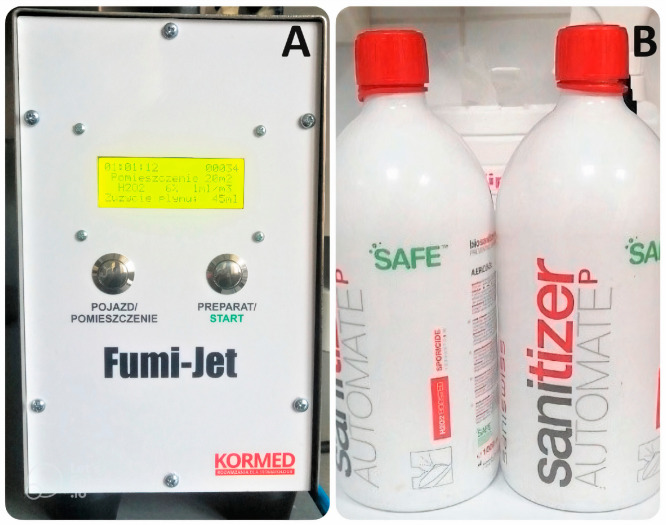
(**A**) The compressed air device (Fumi-Jet, Kormed, Poland). (**B**) Bottles of 6% hydrogen peroxide biosanitizer (Saniswiss, Switzerland).

The office was fumigated using a 6% hydrogen peroxide [1] biosanitizer (Saniswiss, Genève, Switzerland) with a compressed air device (Fumi-Jet, Kormed, Poland). The treatment duration was 20 min, with 3 min allocated for fumigation and 17 min for the waiting period to allow for the chemotoxic effect. During the process, 45 mL of 6% hydrogen peroxide was sprayed. The treated room had a size of 20 m^2^. To verify the effectiveness of the hydrogen peroxide, control strips (Roam Technology (Genk, Belgium)) were placed near the plates with a microbiological base. These strips assessed the correct concentration of hydrogen peroxide necessary to achieve a bactericidal effect in the dental office room (Figure 2).

### 2.6. Statistical Analysis

The data’s normality was assessed using the Kolmogorov–Smirnov test. Total bacterial counts (cfu/m^3^) before and after 6% H_2_O_2_ fumigation were compared using a Student’s *t*-test. The association between the bacterial contamination of the microbiological plates before and after H_2_O_2_ fumigation was analyzed using the Chi-square test. Statistical analyses were conducted using Statistica software (Version 13.3) (StatSoft, Tulsa, OK, USA), considering values below *p* = 0.05 as statistically significant.

## 3. Results

### 3.1. Analyzing the Total Bacterial Counts in the Air of the Dental Office during Caries Treatment

The total bacterial colony (TBC) level, measured in cfu/m^3^, demonstrated a significant decrease in the number of bacteria following room environment fumigation (*p* < 0.001). The TBC after caries treatment, before (G1 group) and after (G2 group) fumigation, was 817.2 ± 208.2 and 163.1 ± 145.7, respectively. Upon comparing both groups, it was evident that the utilization of a 6% H_2_O_2_ room fumigation resulted in an 80% reduction in the total bacterial count (Table 1).

### 3.2. Analyzing the Quality of Bacteria in the Dental Office Air during Caries Treatment

The qualitative analysis of the microbiological assessment of the air after caries treatment indicated the greatest variety of bacterial strains on the plates distributed before H_2_O_2_ fumigation. The predominant bacteria observed in the microbiological plates before fumigation were *Micrococcus* and *Bacillus* species, observed in 80% and 60% of the plates, respectively. Additionally, *Staphylococcus epidermidis* (23%), *Staphylococcus saprophyticus* (20%), *Staphylococcus arlettae* (17%), and *Staphylococcus warneri* (7%) were found. Application of a H_2_O_2_ room fumigation led to a significant reduction in bacterial numbers, amounting to 79.2% for *Micrococcus* species (*p* < 0.001), 83.3% for *Bacillus* species (*p* < 0.001), and 100% for *Staphylococcus arlettae* (*p* < 0.05). For other species, statistically insignificant results were observed (*p* > 0.05) (Table 2).

## 4. Discussion

Maintaining microbiological cleanliness is a crucial aspect of workplace safety in environments at higher risk of pathogenic microorganism infection, such as laboratories, hospitals, health centers, and dental offices [2,11,28,29]. Our study results confirmed that the utilization of a 6% H_2_O_2_ room fumigation resulted in an 80% reduction in the total bacterial count. Therefore, we rejected the null hypothesis stating that there is no significant difference in bacterial counts before and after the implementation of a H_2_O_2_ fumigation. Additionally, the application of a H_2_O_2_ room fumigation led to a significant reduction in bacterial numbers, with a decrease of 79.2% for *Micrococcus* species, 83.3% for *Bacillus* species, and 100% for *Staphylococcus arlettae*. The hydrogen peroxide aerosol is very effective in the elimination or significant reduction of microbial numbers, as demonstrated in studies related to medicine or industry [12,13,30]. Fumigation is a mandatory international requirement, particularly in laboratories with maximum containment measures [30]. However, given its effectiveness, this method can also be applied in other settings, such as hospitals or health centers, particularly during periods of elevated respiratory infections or amid a pandemic [2].

Hydrogen peroxide, used as a fumigant, demonstrates effective decontamination against a broad spectrum of microorganisms [24,31,32,33,34]. Compared to other fumigants, like chlorine dioxide, ethylene oxide, methyl bromide, and formaldehyde, hydrogen peroxide is deemed less toxic [2,35]. Moreover, it undergoes catalytic breakdown into water and oxygen upon neutralization [12]. The main focus of our present study was to evaluate the efficacy of H_2_O_2_ fumigation in a dental office after the caries treatment. The study conducted by Shivakumar KM et al. [36] revealed that atmospheric microbial contamination (measured in CFUs/plate) was four times higher during working sessions compared to the levels before the working sessions. The treatment involving the use of a high-speed rotary instrument significantly increases the level of aerosol particles in the air [16,37,38]. These particles can serve as a source of infection transmission among other patients and dental office personnel [39,40]. In our study, we observed a higher percentage decrease in bacterial numbers after 20 min of H_2_O_2_ fumigation: *Micrococcus* species (79.2%), *Bacillus* species (83.3%), *Staphylococcus epidermidis* (71.4%), *Staphylococcus saprophyticus* (80%), *Staphylococcus arlettae* (100%), and *Staphylococcus warneri* (100%). In contrast to our results, a study by Rogers J. and Choi YW. indicated no bacterial growth (100% reduction in TBCs) for *Francisella tularensis*, *Bacillus subtilis*, *Geobacillus stearothermophilus*, and spore strips (*Bacillus atrophaeus*). The variation in results can be explained by the extended duration of hydrogen peroxide fumigation, which lasted 2 h [12].

The effectiveness of hydrogen peroxide (H_2_O_2_) fumigation is closely tied to the duration of exposure [12,32]. Prolonged exposure allows H_2_O_2_ to thoroughly interact with bacteria, enhancing the likelihood of successful disinfection [24]. However, the exposure duration needs to be balanced with factors such as hydrogen peroxide concentration, environmental conditions (humidity and temperature), bacterial types, the presence of organic materials, and ventilation [11,12]. Various studies, including our present study, have demonstrated substantial microbial reduction in less than 30 min of fumigation [24,32,34]. Heckert RA and colleagues [32] illustrated the effectiveness of reducing virus titers to 0 embryo-lethal doses for avian viruses and less than 10 tissue culture infective doses for mammalian viruses after 30 min of exposure to vapor phase hydrogen peroxide. Additionally, Johnston MD et al. [34] showed complete inactivation of toxigenic *Clostridium botulinum* proteolytic and non-proteolytic strains following 6 and 7 min exposure times to vapor phase hydrogen peroxide. Rogers JV et al. [24] showcased significant decontamination efficacy against spores from *Bacillus anthracis*, *Bacillus subtilis*, and *Geobacillus stearothermophilus* exposed to ≥1000 ppm hydrogen peroxide gas for 20 min. In contrast, French GL et al. [33] found that after exposing six rooms to hydrogen peroxide vapor, only 1.2% of 85 swabs yielded methicillin-resistant *Staphylococcus aureus* (MRSA) after 5 h of H_2_O_2_ fumigation, based on enrichment culture. The laboratory equipment exposed to the gas appeared to suffer no adverse effects [32]. Vapor phase hydrogen peroxide decontamination can be recommended as a safe and efficacious way of removing potentially virus-contaminated objects from biocontainment level III laboratories in which exotic animal disease virus agents are handled [32]. Properly controlling exposure time alongside these variables ensures the optimal bactericidal effect of H_2_O_2_ fumigation in medical settings, emphasizing the critical role time plays in achieving successful disinfection outcomes [32,33].

In the realm of fumigation devices, there exists a diverse array, each employing specific chemical agents for dispersion, including hydrogen peroxide aerosols, chlorine dioxide, and mixtures like peracetic acid and hydrogen peroxide [7,11]. These agents are dispersed through innovative technologies categorized based on the size and dispersion properties of the liquid particles [7]. Hydrogen peroxide aerosol generators, functioning with concentrations of 3–7%, are characterized by their effectiveness in bactericidal and virucidal actions, particularly on surfaces and objects. In contrast, hydrogen peroxide dry mist generators break down particles to about 5 μm, enhancing their efficacy against viruses and bacteria in both air and surface environments [1,13,41,42]. Moreover, these devices come in different builds, with turbo foggers utilizing high-speed turbines for efficient aerosolization and compressed air foggers utilizing compressors for controlled disinfectant dispersion [7]. Each type has its advantages, whether it is the excellent mist quality of the compressed air foggers or the effective room coverage of the turbo foggers. These advancements reflect a nuanced approach to room fumigation, catering to various needs in medical environments [12,13,23,42].

The effectiveness of the fumigation process, as well as the percentage concentration of the chemical, depends on the quality of the fog generated by the fumigation devices [11,12]. A pilot study by Matys et. al. [23] using a compressed air device demonstrated that fumigation with 6% chemically stabilized hydrogen peroxide allows the sprayed steam to effectively reach a high concentration of disinfectant in various places in the dental office, including open drawers of office cabinets, floor areas, and window sills, as well as around the assistant in the central area of the office. The dark coloration observed in the tests confirmed the correct concentration of sprayed hydrogen peroxide in the tested areas of the office. In the current study, 6% plasma H_2_O_2_ was utilized for fumigation, a choice recommended for its bactericidal and virucidal properties, effectively decontaminating the air and surfaces of the dental office [1]. The process of hydrogen peroxide plasmaization enables its concentration to be maintained in the air and on surfaces for several minutes to ensure the eradication of microorganisms. An alternative method involves the chemical stabilization of hydrogen peroxide, ensuring the maintenance of a proper concentration after spraying and allowing the disinfectant to be stored for several months without significant decomposition [1,43].

Microbiologists commonly employ culturing techniques to analyze the content of aerosols [44]. In this method, bacteria from a sample are cultivated on specific media, including both solid and liquid mediums, to facilitate the formation of colonies [9]. The selection of Columbia Agar with 5% Sheep Blood as the microbiological medium in our study was deliberate and based on its specific properties conducive to our research objectives. Columbia Agar provides a nutrient-rich environment that supports the growth of a wide range of microorganisms, making it suitable for the cultivation of bacteria encountered in dental settings [45]. The addition of 5% Sheep Blood further enhances its utility by providing essential nutrients and fostering the growth of fastidious bacteria that may be present in aerosol samples [45]. This medium is particularly well suited for detecting and isolating various bacterial species, including those commonly found in dental biofilms [46]. Moreover, the incorporation of blood enriches the medium with factors essential for the expression of hemolysis, aiding in the identification and differentiation of bacterial strains [47].

Last but not least, in addressing the importance of the assessed microbial species in our study, it was crucial to recognize their potential implications for the development of infections within dental settings [48]. While our focus was predominantly on controlling aerobic bacteria, it was noteworthy that anaerobic bacteria played a significant role in the origin of various dental diseases, as supported by existing evidence [49]. The assessment of specific bacterial species found in the air of the dental office in our present study is important for understanding and addressing the potential development of infections. Each of these bacterial species has unique characteristics and roles in microbial communities. *Micrococcus* and *Bacillus* species, for instance, are commonly found in various environments, including soil, water, and air [50]. Recognizing these bacteria as potential sources of contamination is crucial for implementing targeted infection control measures [49,50]. *Staphylococcus* species, including *S. epidermidis*, *S. saprophyticus*, *S. arlettae*, and *S. warneri*, are associated with the human skin microbiota and may be introduced into the dental environment during patient interactions [51]. Identifying and studying these bacterial species is essential because they may serve as potential sources of contamination and contributors to the risk of infections in dental settings. Understanding their prevalence, abundance, and dynamics allows for targeted preventive and control measures [51]. Additionally, recognizing the presence of specific Staphylococcus species is particularly relevant due to their association with skin and potential links to nosocomial infections [52,53].

This study was subject to several limitations. Firstly, the total bacterial count (TBC) in the office air was measured using the conventional Koch sedimentation method, which is known for its limited accuracy. To enhance precision, more advanced microbial analysis techniques such as polymerase chain reaction (PCR), next-generation sequencing (NGS), and metagenomics should be considered in future research. Additionally, there is a need for investigations to understand the influence of dental procedures like root scaling or dental sandblasting on the bactericidal and virucidal effects of hydrogen peroxide fumigation. In addition to highlighting the efficacy of hydrogen peroxide (H_2_O_2_) fumigation in lowering bacterial counts within a dental office environment, it is crucial to acknowledge certain limitations. One noteworthy limitation is the lack of assessment of the antiviral and antifungal effects of hydrogen peroxide. The study also falls short in providing a direct comparison with alternative methods of air decontamination. Addressing these aspects in future research endeavors would offer a more comprehensive evaluation of sterilization and decontamination practices in dental settings.

## 5. Conclusions

Our study showed the significant impact of hydrogen peroxide (H_2_O_2_) fumigation on bacterial contamination within dental office environments. The substantial decrease in aerobic bacteria concentration, indicated by the notable reduction in total bacterial colony levels post-fumigation, affirms the effectiveness of this approach. While certain strains exhibited statistically insignificant changes, the overall pattern highlights the precision of the method. The implementation of this technique could substantially reduce the risk of infections in dental offices, creating a healthier environment for both healthcare providers and patients.

## Figures and Tables

**Table 1:** Mean results of total bacteria colony level before and after H_2_O_2_ fumigation.

Groups	Student *t*-test; df 58; *n* = 60			
	*n*	Mean (cfu/m^3^)	SD	*p* Value
TBC Before H_2_O_2_ Fumigation (G1)	30	817.2	208.2	*p* < 0.001
TBC After H_2_O_2_ Fumigation (G2)	30	163.1	145.7	

cfu/m^3^—colony-forming units per cubic meter; SD—standard deviation; df—degrees of freedom; *n*—number of microbiological plates.

**Table 2:** Reduction in bacterial levels after H_2_O_2_ fumigation. The table displays the frequency of occurrence of a specific bacterial strain per the total number of microbiological plates used in the groups before and after fumigation.

Bacterial Species	Before Fumigation (G1, *n* = 30)	After Fumigation (G2, *n* = 30)	Contamination Reduction Percentage	*p* Value
*Micrococcus* species	24 (80%)	5 (17%)	79.2%	*p* < 0.001
*Bacillus* species	18 (60%)	3 (10%)	83.3%	*p* < 0.001
*Staphylococcus epidermidis*	7 (23%)	2 (7%)	71.4%	*p* > 0.05
*Staphylococcus saprophyticus*	5 (20%)	1 (3%)	80%	*p* > 0.05
*Staphylococcus arlettae*	5 (17%)	0 (0%)	100%	*p* < 0.05
*Staphylococcus warneri*	2 (7%)	0 (0%)	100%	*p* > 0.05

## Data Availability

Data is contained within the article.
